# The Book World of Medicine and Science

**Published:** 1895-01-19

**Authors:** 


### THE BOOK WORLD OF MEDICINE AND SCIENCE.

MYXÆDEMA, CRETINISM, AND THE GOITRES. By EDWARD T. BLAKE, M.D. (Bristol: John Wright and Co. 1894. Pp. 82. Price 3s. 6d.)

This is a book of hypotheses, and to those whose minds are set upon positive knowledge will be disappointing. Yet it is not uninteresting, and, although it would be idle to pretend that the author proves his thesis, the book is strongly suggestive not only of its correctness but of the possibility of its being applicable to many other conditions besides those formally included. The difficulty, in fact, in such a matter is to know where to stop.

The points sought to be established are: (1) That Graves's disease in woman is an autotoxis, most frequently caused by the absorption of purulent products; that process being aided rather than induced by the toxines of terror or shock. (2) Similar products lead to the production of rheumatism in males, by acting in the same locality, viz., the medulla oblongata. (3) If these products abolish the functions of the thyroid, we may get myxædema. (4) If they invade the cortex, in large quantity and abruptly, we get a psychosis, as, for example, mania; or some neurosis, such as epilepsy. (5) If the invasion be more gradual, and the poisons be more diluted, we get chorea. This is, indeed, the apotheosis of the toxins, which certainly just at present are having their day. Nevertheless there are many facts, and still more arguments, pointing to the probability of many maladies being set up by a process of auto-intoxication. If delirium and tremor can be set up by the toxins produced in the fever process acting on nerve and muscle cells, and if they can also be caused in a more chronic form by more prolonged poisoning by that very toxic substance, alcohol, who is to say that mania or melancholia may not be produced by some vile toxins brewed by bad livers or some other phase of deficient metabolism? This is humoral pathology with a vengeance. The difficulty is to prove it, and, if proved, to see where the influence is to end. At the same time disproof is almost as hard to find. The positive knowledge we possess on most subjects is so small that an author may well be excused an occasional flight into hypothesis, especially when he honestly calls it by its name, and presents it in such good type and paper as in the book before us.

CATALOGUE OF PATHOLOGICAL SPECIMENS IN THE MUSEUM OF GUY'S HOSPITAL. Third edition, vol. I. By LAURISTON E. SHAW, M.D., and E. COOPER PERRY, M.A., M.D. (London: J. and A. Churchill. 1894.)

Following in the wake of St. Bartholomew's, St. Mary's, and other London hospitals, the authorities at Guy's have made a great effort to produce a catalogue of their pathological collections, which shall be up to date and abreast of modern descriptive pathology. The task has been entrusted to Dr. Shaw and Dr. Perry, and although to a certain extent they have had the guidance of older catalogues published in 1829 by Dr. Hodgkin, and in 1863 by Drs. Wilks and Habershon, it is a doubtful question whether these worthies have not rather hindered than furthered their efforts. The nomenclature of pathological conditions has undergone almost a complete revolution since the first catalogue was published, and advances in microscopy have enabled pathologists of the present day to correct the diagnosis of their predecessors.

The work has entailed a large amount of section cutting, and the plan of adding a short account of the clinical history of the case illustrated by the specimen, has necessitated an enormous amount of patient reference to clinical reports in the archives of the hospital. All honour is due to the two pathologists who have thus completed the first volume, and the work reflects credit not only on themselves but on the whole hospital. The authorities at St. George's and at one or two other hospitals at which the pathological catalogues are still chaos, may learn a useful lesson by a glance at the pages of this work.

